# Cylindrical Layered Bone Scaffolds with Anisotropic Mechanical Properties as Potential Drug Delivery Systems

**DOI:** 10.3390/molecules24101931

**Published:** 2019-05-19

**Authors:** Maria Francesca Di Filippo, Sofia Amadori, Sonia Casolari, Adriana Bigi, Luisa Stella Dolci, Silvia Panzavolta

**Affiliations:** 1 Department of Chemistry “G. Ciamician”, University of Bologna, Via Selmi 2, 40126 Bologna, Italy; maria.difilippo5@unibo.it (M.F.D.F.); sofia86@live.it (S.A.); sonia.casolari@unibo.it (S.C.); adriana.bigi@unibo.it (A.B.); silvia.panzavolta@unibo.it (S.P.); 2 Department of Pharmacy and BioTechnology, University of Bologna, Via S. Donato 19/2, 40127 Bologna, Italy

**Keywords:** gelatin, scaffold, tissue regeneration, biomimetic, strontium release

## Abstract

3D cylindrical layered scaffolds with anisotropic mechanical properties were prepared according to a new and simple method, which involves gelatin foaming, deposition of foamed strips, in situ crosslinking, strip rolling and lyophilization. Different genipin concentrations were tested in order to obtain strips with different crosslinking degrees and a tunable stability in biological environment. Before lyophilization, the strips were curled in a concentric structure to generate anisotropic spiral-cylindrical scaffolds. The scaffolds displayed significantly higher values of stress at break and of the Young modulus in compression along the longitudinal than the transverse direction. Further improvement of the mechanical properties was achieved by adding strontium-substituted hydroxyapatite (Sr-HA) to the scaffold composition and by increasing genipin concentration. Moreover, composition modulated also water uptake ability and degradation behavior. The scaffolds showed a sustained strontium release, suggesting possible applications for the local treatment of abnormally high bone resorption. This study demonstrates that assembly of layers of different composition can be used as a tool to obtain scaffolds with modulated properties, which can be loaded with drugs or biologically active molecules providing properties tailored upon the needs.

## 1. Introduction

The main requirements of advanced materials for regenerative medicine are a hierarchical 3D architecture, a friendly interaction with biological tissues and promotion of tissue repair.

For large bone defects, bulk scaffolds are capable of filling the missing segments and provide sufficient mechanical support for host bone immediately after implantation [1]. Therefore, they are preferred to other forms of materials (such as cements, injectable gels and membranes) for large bone defects [2].

Since native bone is a complex material with well-designed architecture, its successful integration and regeneration require that bone scaffolds support bone metabolism and therefore be reabsorbed in situ, allowing the growth of the new tissue.

Three-dimensional scaffolds mimicking the structure of extracellular matrix (ECM) provide the necessary support for cell attachment, growth and differentiation and define the overall shape of a bone tissue engineered transplant [3]. Inspired by the constituents of natural bone, a number of studies have used organic−inorganic compounds to fabricate bone-repairing scaffolds [4,5,6]. To this aim, various fabrication techniques, such as porogen leaching, gas foaming, phase separation, fiber meshing, supercritical fluid processing, microsphere sintering, 3D printing and freeze-drying, have been employed [7,8]. Among different materials proposed for bone scaffolds composition [9,10], collagen and its derivative gelatin attracted great interest, mainly due to their chemical similarity to organic phase of bone tissue [11,12]. Gelatin is obtained by thermal denaturation or physical and chemical degradation of collagen through the breaking of the triple- helix structure into random coils [13,14]. Gelatin is not antigenic under physiological conditions, it is biodegradable and biocompatible and these properties support its wide employment in pharmaceutical and medical fields, in tissue engineering, wound dressing and gene therapy [15,16]. Furthermore, it is completely resorbable in vivo, and its physicochemical properties can be suitably modulated; however, due to its high solubility in aqueous environment, a crosslinking reaction is mandatory [17].

In this study we prepared 3D rolled scaffolds for bone tissue engineering using gelatin. The 3D rolled scaffolds were stabilized by crosslinking with genipin, a natural crosslinking molecule extracted from the fruits of *Gardenia jasminoides* Ellis, which exhibits low cytotoxicity when compared to other chemical crosslinking agents. Genipin can efficiently crosslink cellular tissues and biomaterials containing primary amino-groups to form blue pigments [18,19,20,21]. We utilized genipin solutions at different concentrations in order to obtain strips at different content of cross-linker and hence, with different life-times. Furthermore, we investigated the possibility to enrich the material with an inorganic phase: enrichment with strontium substituted hydroxyapatite (Sr-HA) was performed in order to obtain systems with improved mechanical performances and with an anti-osteoporotic activity, thanks to a sustained release of strontium over time. Strontium is known to display a beneficial role on bone remodeling and it has been proposed for the treatment of pathologies associated to excessive bone resorption (e.g. osteoporosis) [22,23]. Its action on bone turnover has been ascribed to the combined effect of decrease of bone resorption and enhancement of bone formation [24,25], and clinical studies show a beneficial effect of strontium treatment in osteoporotic patients [26,27]. These positive results have stimulated a growing interest toward calcium phosphates bioceramics, coatings, cements and scaffolds as possible delivery systems of Sr ions [28,29,30,31,32,33,34].

The structure of the scaffold plays a critical role in the reconstruction of bone tissues but conventional porous scaffolds exhibit isotropic transmission of stress [35], which hampers their integration with the anisotropic host bone. Accordingly, finely designed 3D scaffolds with similar structures of natural bone are required for better bone integration and even for successful osteointegration and functional reconstruction of bone defects: in fact, it has been demonstrated that that spiral- cylindrical arrangement of hybrid chitosan/cellulose/nano-hydroxyapatite membranes can promote complete infiltration of bone tissues in vivo [36]. Hence, the rolling-up strategy has been applied by several groups in scaffold fabrication, also made by nanofibres, [37,38,39,40] with the purpose to increase the surface to volume ratio for cell attachment and form a 3D non-planar cell matrix [41].

Herein, we developed new anisotropic gelatin scaffolds, assembling strips at different genipin concentrations, which were curled concentrically to generate a cylindrical scaffold with a spiral cross-section, which composition and structure mimic the osteon concentric arrangement. This approach provides highly porous multi-layered scaffolds with anisotropic mechanical properties, which should widen the range of possible applications. On this basis, porous 3D rolled gelatin scaffolds containing 30% (*w*/*w*) of Sr-HA were prepared and characterized.

## 2. Results

**Scheme 1:**
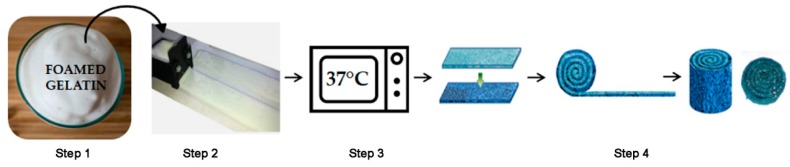
Rolled scaffold preparation.

Cylindrical scaffolds were prepared by rolling up gelatin layers as depicted in Scheme 1. The use of doctor-blade allows to obtain regular stripes of foamed gelatin (step 2) with a predetermined width and thickness. Table 1 reports the composition and the label of each scaffold. The novel approach developed in this paper provides highly porous multi-layered scaffolds with anisotropic mechanical properties. 

### 2.1. Sr-HA Characterization

It is well known that strontium is present in the mineral phase of the bone, especially at the regions of high metabolic turn-over [42], and that its administration to post-menoapausal women has beneficial effect in the treatment of osteoporosis, reducing bone resorption and enhancing bone formation. Strontium can easily replace calcium in the hydroxyapatite structure, because of the chemical similarity between the two cations. 

**Figure 1:**
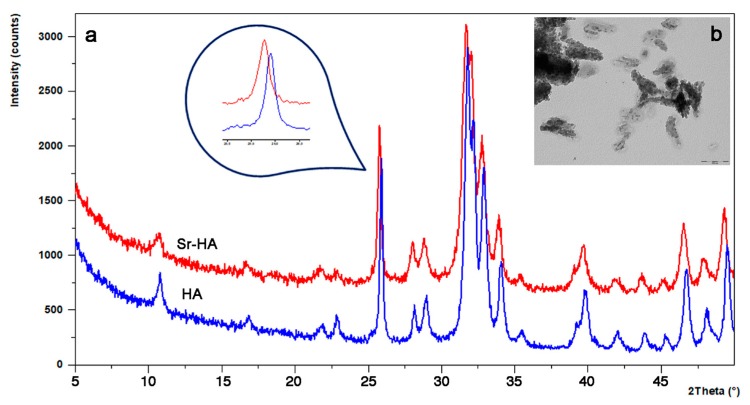
(**a**) X-ray diffraction pattern of Strontium-substituted hydroxyapatite (Sr-HA) and Hydroxyapatite (HA). The inset reports an enlargement of the 25–26.5 2θ range where the shift of the 002 reflection is clearly appreciable; (**b**) TEM image of Sr-HA nanocrystals (bar = 100 nm).

With this aim, Sr-substituted hydroxyapatite, with a Sr content of 10% (in atoms), was synthesized and employed for scaffold manufacturing. The X-ray powder diffraction pattern of the obtained Sr-HA is reported in Figure 1a together with the pattern of hydroxyapatite (HA) as comparison. 

All the peaks in Sr-HA pattern are characteristic of hydroxyapatite, which is the only crystalline phase obtained from the synthesis. The incorporation of Sr into the crystal lattice of hydroxyapatite is confirmed by the shift of the main diffraction peaks of Sr-HA, at lower angles when compared to those present in the X-ray diffraction pattern of the samples synthesized in the absence of strontium (HA), as reported in literature [28]. This shift is in agreement with the substitution of the bigger strontium ion to calcium into the hydroxyapatite structure [28]. Sr-HA appears constituted of nanocrystals with small dimensions (about 30 nm long) and an irregular shape, as it can be seen in the TEM image (Figure 1b).

### 2.2. Scaffold Characterization

**Figure 2:**
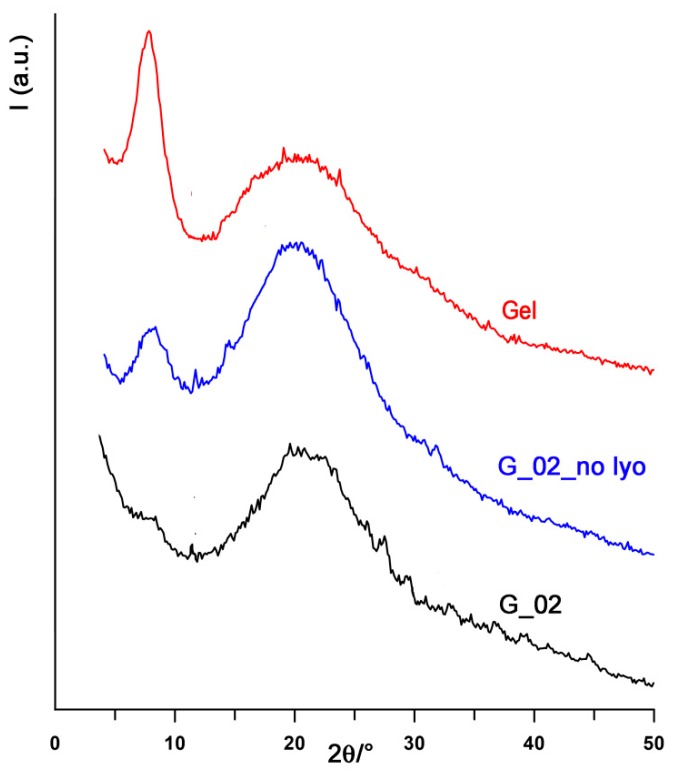
X-rays powder diffraction patterns recorded on gelatin foam (Gel), gelatin foam containing genipin before lyophilization (G_02_no lyo) and the same sample after lyophilization (G_02).

The preparation of the scaffolds implies gelatin foaming, crosslinking and air drying. The structural modifications of gelatin induced by this procedure was analyzed using X-ray diffraction analysis. In fact, the wide-angle X-ray diffraction peak of collagen at about 1.1 nm, which is related to the diameter of the triple helix, can be utilized as an index of the degree of renaturation of gelatin [43]. Figure 2 reports the wide-angle X-ray diffraction patterns of the samples during the different steps of the preparation of the scaffolds. All the patterns display a diffraction peak at about 8° of 2θ, corresponding to the periodicity of 1.1 nm, and a broad halo centered around 20° of 2θ corresponding to a periodicity of about 0.45 nm, due to the distance between adjacent polypeptide strands [44]. The 1.1 nm peak displays a high relative intensity in the pattern recorded from gelatin foam (Gel). The sample obtained after foaming in presence of genipin before lyophilization (G_02_no lyo) exhibits an X-ray diffraction pattern where the relative intensity of the 1.1 nm peak appears reduced, suggesting a decrease of the triple helix content. Further reduction is obtained after freeze-drying (G_02), as it results from the comparison of the X-ray diffraction patterns reported in Figure 2. 

**Figure 3:**
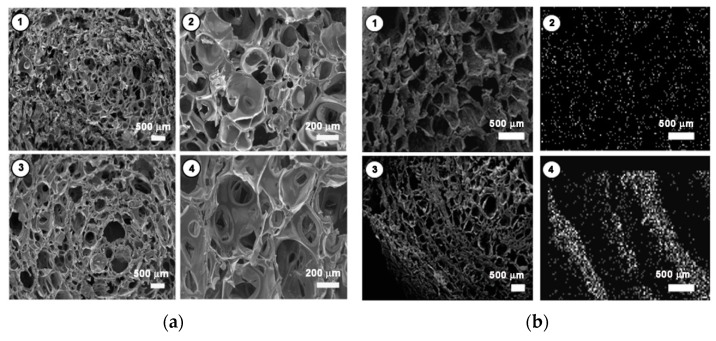
SEM images of samples: (**a**) single-layer curled scaffolds G_0.1 (1, 2) and G_0.15 (3, 4); (**b**) flat layer scaffold S_0.1_Sr-HA (1) and double-layer rolled scaffold G_0.1_Sr-HA/G_0.2 (3) with their EDS map showing the presence of Sr (2 and 4, respectively).

The morphology of the obtained scaffolds was investigated by means of scanning electron microscopy. The images in Figure 3a, related to single-layer rolled scaffolds, confirm the high porosity and the interconnection of pores; furthermore, the layers appear strictly joined, forming a continuous network without uneven areas at the joint points. Figure 3(b1) shows the structure of a single flat layer (S_0.1_Sr-HA) containing Sr-HA: the homogeneous distribution of Sr-HA nanocrystals inside the gelatin network is confirmed by the EDS maps reported in Figure 3(b2), where the small bright zones indicate the presence of Strontium.

The morphology of the double layered scaffold G_0.1_Sr-HA/G_0.2 is showed in Figure 3(b3), together with its EDS map (Figure 3(b4)): the layered structure is well evident, since the layers containing the inorganic phase appear more compact. These images clearly show that rolling up layers containing Sr-HA provokes a reduction of the pores dimensions in comparison with those of the flat layer (compare Figure 3(b3) with Figure 3(b1)). Furthermore, the Sr-HA containing layers can be easily identified by the EDS map (Figure 3(b4)), which verifes the presence of strontium inside the cylindrical structure.

### 2.3. Mechanical Properties

**Scheme 2:**
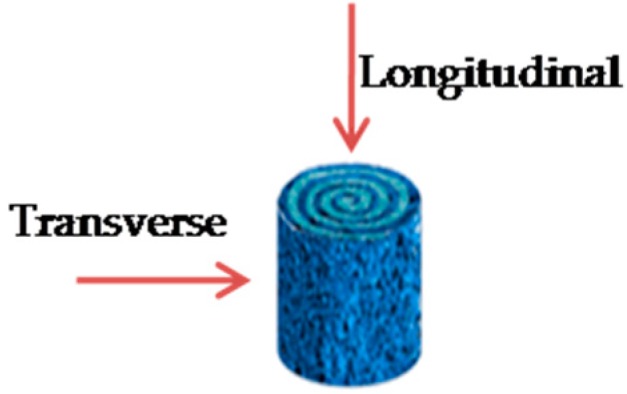
Longitudinal and transverse directions for mechanical tests.

Mechanical properties were measured under compression both in longitudinal and transverse direction (Scheme 2). 

**Figure 4:**
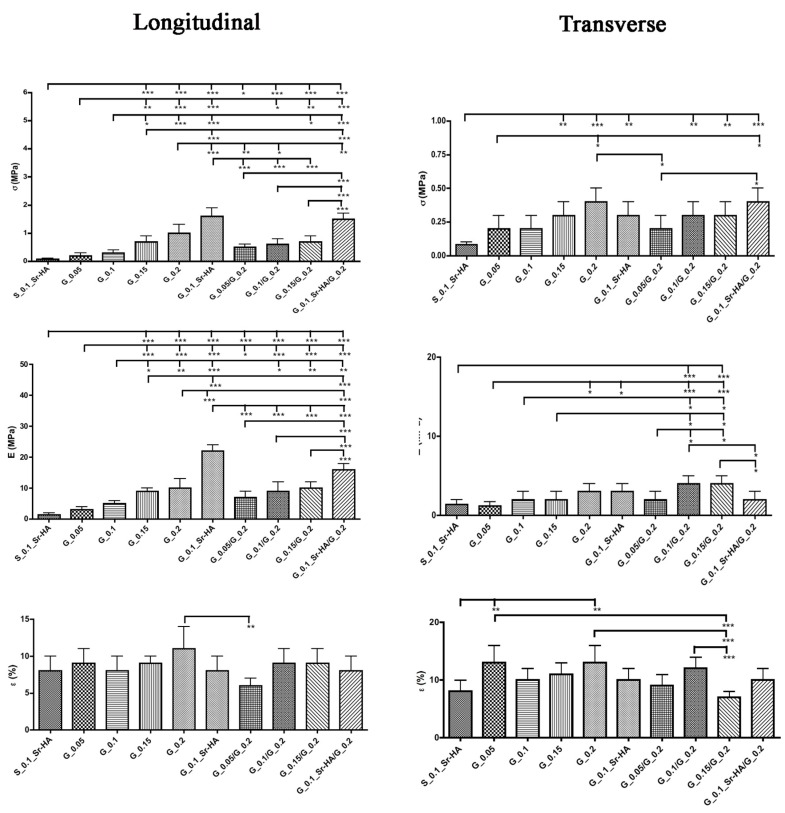
Mechanical properties in compression, measured in longitudinal and transverse direction. Values of maximum stress at break (σ), Young’s modulus (E) and elastic deformation (ε) are reported. Each value corresponds to the average of 6 determinations and it is reported with its own standard deviation and statistical analysis. Significant differences (one-way ANOVA) in σ (MPa), E (MPa) and ε. Asterisks (* *p* < 0.05, ** *p* < 0.01 and *** *p* < 0.001) indicate results of post-hoc test (Tukey).

The experimental curves were used to calculate the maximum stress at break (σ), determined via linear regression of the initial linear regime of the stress-strain curves, the elastic deformation (ε%), evaluated at the point of transition from linear to collapse regime, and the Young’s modulus (E). The mean values obtained for the rolled scaffolds cross-linked with different genipin concentrations are reported in Figure 4. Data reveal the anisotropic behavior of the samples, which exhibit better mechanical properties when compressed along the longitudinal direction (parallel to the long axis of the cylindrical scaffold). Only the flat single-layer S_0.1_Sr-HA shows an isotropic behavior when tested longitudinally and transversely. An improvement of the mechanical properties, especially along the longitudinal direction, can be observed by increasing the concentration of the crosslinking agent, which provokes an increase of both the values of maximum stress at break and of Young’s modulus, in agreement with previous data [45]. Moreover, the scaffolds prepared from two different layers exhibit mechanical properties intermediate between those of the single layer scaffolds. The compaction caused by rolling the scaffolds enhances the mechanical properties, as it results from the comparison of the values of the mechanical parameters of S_0.1_Sr-HA with those of G_0.1_Sr-HA. The presence of the inorganic phase significantly strengthens the scaffolds: the stress at break of G_0.1_Sr-HA measured in longitudinal direction is five times higher than that of G_0.1 (1.6 ± 0.3 for G_0.1_Sr-HA and 0.3 ± 0.1 for G_0.1), whereas the values of the mechanical parameters along the transverse direction do not show significant variation as a function of composition. 

### 2.4. Extent of Crosslinking

The extent of crosslinking, calculated for single-layer rolled scaffolds and expressed as stated in Equation (1) is reported in Table 2. It can be inferred that crosslinking significantly increases from G_0.05 to G_0.2 in agreement with the increase of genipin concentration used for the preparation of the scaffolds.

### 2.5. Water Uptake Ability (WUA)

The water uptake ability of the samples is calculated by means of Equation (2): when the composition is enriched with Sr-HA, the values have been normalized to the weight of gelatin alone. The obtained values, reported in Table 3, highlight that the amount of adsorbed PBS depends on the number of layers used to prepare the scaffolds. Indeed, the double-layered samples display WUA values higher than those of single-layer scaffolds (e.g., G_0.1/G_0.2 vs. G_0.1 or G_0.15/G_0.2 vs. G_0.15), most likely because of the presence of uneven areas between different layers which provide more space for the solution uptake. On the other hand, the presence of Sr-HA inside the scaffolds does not seem to influence significantly the WUA.

### 2.6. Gelatin Release

Average values of the amount of gelatin released from the samples after immersion in NaCl 0.9% at 37 °C are reported in Table 4. 

The higher the concentration of genipin used to crosslink the scaffold, the longer the life-time of the material: the scaffold with the highest percentage of genipin achieved a strong stability and it does not dissolve even after 70 days, while the dissolution of samples cross-linked with lower genipin concentrations starts already at 14 days, in agreement with previous data [40].

### 2.7. Strontium Release

**Figure 5:**
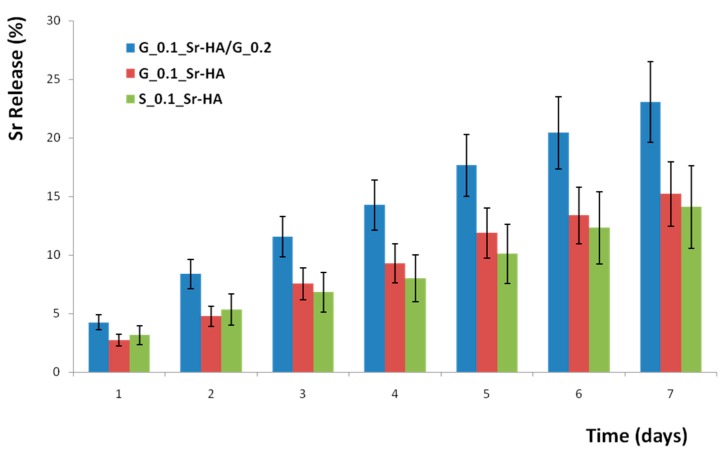
Strontium cumulative release from samples G_0.1_Sr-HA/G_0.2 (blue), G_0.1_Sr-HA (red) and S_0.1_Sr-HA (green) as a function of soaking time in NaCl 0,9% at 37 °C.

The amount of strontium released as a function of soaking time in NaCl 0.9% at 37 °C was evaluated by means of atomic absorption spectrophotometric analysis. The results reported in Figure 5 show that after seven days strontium cumulative release reaches values of about 23% for the double-layered rolled scaffolds, 16% for the single-layer rolled scaffolds and 14% for the flat layer, following the same trend as that of the water uptake ability results. Hence, the greater amount of solution that the double-layered rolled scaffolds are able to absorb could be responsible for the greater strontium release in solution. 

## 3. Discussion

Porous gelatin scaffolds can be obtained through foaming and freeze-drying gelatin aqueous solutions [46,47,48]. Herein, after foaming, we applied doctor-blading, a technique in which a blade with a certain gap height is used to spread a viscous solution [49], to produce porous large gelatin strips, which were then stabilized using genipin as crosslinker. Gelatin is obtained through collagen denaturation implying the breaking of the triple helix structure, which is partially recovered during gelatin gelling. The presence of genipin during the cooling of the foam involves the formation of crosslinks which inhibits the conformational disorder-order transition, as shown by the decrease of the relative intensity of the 1.1 nm diffraction reflection after gelatin foaming in the presence of genipin (Figure 2). The further reduction of the relative intensity of the diffraction peak observed on the scaffolds after freeze drying is consistent with a further decrease of the triple helix content, in agreement with what previously reported for wet and freeze dried collagen [50].

Cylindrical layered scaffolds obtained through curling gelatin strips on themselves displayed high porosity, although slightly less than that of the flat layer because the rolling up provokes shrinkage of the pores. At variance with the great majority of the porous scaffolds proposed for bone repair [4], the peculiar three-dimensional structure of the scaffold resulted in anisotropic mechanical properties. Both the values of maximum stress at break and of elastic modulus in compression were significantly higher when measured along the longitudinal than the transverse direction of the scaffolds. Moreover, also the values measured in the transverse direction were higher than those measured for the flat-layer scaffold, which showed isotropic mechanical properties. The mean values of mechanical properties are in the range reported for normal cancellous bones [51], which also display anisotropic behavior [52,53]. It follows that the rolled scaffolds developed in this work could find useful applications in defects of trabecular bone tissue.

Furthermore, the mechanical properties could be modulated through variation of the concentration of the crosslinking agent: increasing of genipin concentration up to 0.2% (*w*/*w* of genipin with respect to the amount of gelatin) provokes an increase in the degree of crosslinking up to about 30% and, as a consequence, an improvement of the mechanical properties. Crosslinking also modulates scaffold stability, as reported in Table 4. The extremely versatile method employed here allows to prepare composite multi-layered scaffolds in a very simple way. In fact, composite scaffolds were obtained by rolling up two overlapped layers at different degree of crosslinking. Morphological investigation showed that the double-layered curled scaffolds present a clear separation between the layers, which could be responsible for the greater water uptake ability of these materials in comparison with those measured for the single-layer rolled scaffolds. These gaps are very important for an improved nutrient transport within the matrix because they could meet the nutrient requirement of the inner regenerated tissue which is subject to the restrained diffusion rate of oxygen and other nutrient [41]. On the other hand, the separation between the layers does not seem to influence the mechanical properties, which are consistent with those characteristic of the two different layers. The introduction of Sr-HA in the composition of the scaffolds provoked a further enhancement of the mechanical properties, in agreement with what previously reported for isotropic scaffolds which showed increasing values of stress at break and Young modulus on increasing the inorganic phase content [54]. The scaffolds exhibit a strontium sustained release, which is modulated by the amount of water absorption. The release can be modulated by varying the layer where the drug is loaded or loading the drug in both layers, as well as increasing the gap between layers, indicating that these scaffolds can be utilized as systems for the local treatment of pathologies characterized by abnormally high bone resorption. Furthermore, the approach followed in this work to obtain cylindrical layered scaffolds allows the incorporation of different biologically relevant molecules, ions or drugs in different layers, thus forming a composite system able to deliver active principles with proper tailored kinetics.

## 4. Materials and Methods 

### 4.1. Materials

Type A gelatin from porcine skin (Sigma Aldrich, St. Louis, MO, USA) and genipin (Wako Chemicals, Osaka, Japan) were used. Glycine was purchased from Sigma Aldrich. Strontium-substituted hydroxyapatite (Sr-HA) synthesis was performed through direct synthesis in aqueous solution. Briefly, 50 mL of 0.65 M (NH_4_)_2_HPO_4_ solution (pH 10 adjusted with NH_3_) was added dropwise under stirring to the 1.08 M cationic solution Ca(NO_3_)_2_⋅4H_2_O + Sr(NO_3_)_2_, with a Sr/(Ca + Sr) ratio of 0.10 and heated at 90 °C for 4 h. The precipitate was centrifuged, dried at 37 °C, finely ground in a mortar and sieved <80 μm before use.

### 4.2. Scaffolds Preparation

Gelatin (3.75 g) was dissolved in distilled water (32.5 mL) at 55 °C under magnetic stirring. The obtained solution was foamed for 90 seconds before adding genipin solution (2.5 mL, Step 1). Based on the desired life-time of the scaffold, different concentrations of genipin solution were used: 0.05, 0.1, 0.15 and 0.2% (*w*/*w* of genipin with respect to the amount of gelatin) in distilled water. PBS (1 M at pH 7.4, 2.5 mL) were added to accelerate the cross-linking reaction. The foam was then shaped in strips with a uniform thickness of 1 mm and length of 18 cm using a doctor-blade (Step 2) [55]. Doctor-blade used here was fabricated in house, by using a 3D printer. The strips were kept at 37 °C for 1 h in order to start the cross-linking reaction which lead to a change of the scaffold’s color from white to blue (Step 3). Cylindrical single- and double-layer scaffolds with a spiral cross-section were obtained by hand rolling a single gelatin stripe and two overlapped gelatin stripes of different composition, respectively. The stripes were rolled while still damp.

The obtained scaffolds were immersed in glycine 0.1 M for 30 min, washed with distilled water, immersed in ethanol for 24 h and then freeze-dried at −44 °C and 0.1 mbar overnight. The obtained scaffolds were labeled according to their genipin content: G_0.05, G_0.1, G_0.15, G_0.2 (Step 4).

In the case of double-layer scaffolds, the outer layer was always that cross-linked with the major genipin concentration (G_0.2); overlapping stripes with different genipin content allows to obtain the following samples: G_0.05/G_0.2, G_0.1/G_0.2 and G_0.15/G_0.2.

In order to evaluate the scaffolds’ ability to be loaded with drugs, active molecules or inorganic phases and then to release them, the composition of G_0.1 strips was enriched with 30% of Sr-HA (% wt of Sr-HA with respect to the total weight of Sr-HA and gelatin). The inorganic phase was added to gelatin suspension before foaming. Some strips were curled as previously described, obtaining samples labeled as G_0.1_HA, while some others were superimposed and rolled with the G_0.2 stripes in order to obtain double-layer samples labeled as G_0.1_HA/G_0.2. Non-curled strips of the same composition as G_0.1_HA were used as control and labeled S_0.1_HA.

### 4.3. X-ray Powder Diffraction

X-ray diffraction analyses were carried out by means of an X’Pert powder diffractometer (Philips, Eindhoven, The Netherlands) equipped with a graphite monochromator in the diffracted beam. CuKα radiation (λ = 15,418 Å; 40 mA; 40 kV) was used. The diffraction patters had been obtained in the 3–50°/2θ range using a 0.03 step and a 3°/min speed. The qualitative analysis was comparative to diffraction patterns contained in the ICDD Database (website: www.icdd.com).

### 4.4. Morphological Characterizations

Morphological and micro-structural analyses were performed with a Philips XL-20 scanning electron microscope (SEM) operating at 15 kV. Before analysis, the scaffolds were sputter-coated with gold. Energy dispersive X-ray spectrometry (EDS) maps were performed on composite scaffolds using a Philips XL-20 Scanning Electron Microscope operating at 15 kV. Transmission electron microscopy (TEM) investigation of the synthesized powders was carried out using a Philips CM 100 transmission electron microscope operating at 80 kV. A small amount of powder was dispersed in ethanol and submitted to ultrasonication. A drop of the suspension was transferred onto holey carbon foils supported on conventional copper micro grids.

### 4.5. Mechanical Properties

Mechanical characterization of the scaffolds was performed on cylindrical samples in dry conditions. Compression tests were carried out using a 4465 Instron testing machine, equipped with a 1 kN load cell. Six samples were tested for each composition, at a loading rate of 1.0 mm∙min^−1^. Statistical analysis was performed with the Student *t*-test considering a *P*-value of less than 0.05 to be significantly different.

### 4.6. Extent of Crosslinking 

The measurement of the extent of cross-linking is based on the determination of the unreacted ε-amino groups belonging to lysine residues. TNBS (trinitro-benzen sulfonic acid) was used to determine this number, following the procedure previously reported [14]. Briefly, selected samples were allowed to react with TNBS and, after hydrolysis in HCl 6 N, the absorbance of the solution was measured at 346 nm against a blank. The moles of ε-amino groups per gram of gelatin are obtained by the following formula:

(1) $$\mathrm{Moles}\;\mathrm{of}\;\varepsilon –\mathrm{amino}\;\mathrm{groups}/g\;\mathrm{of}\;\mathrm{gelatin}\;=\;\frac{2\cdot A\cdot V}{\varepsilon \cdot b\cdot x}$$

where *A* is the absorbance value at 346 nm, *V* is the final volume of the sample (equal to 0.02 L), *ε* is the molar absorbivity equal to 1.46 × 10^4^ L/mol∙cm, *x* is the optical path of the value of 1 cm and *b* is the weight of the sample under test, expressed in grams. The degree of cross-linking (%) was determined from the ratio between the moles of free *ε*–amino groups in genipin treated gelatin and those in non-crosslinked gelatin.

### 4.7. Water Uptake Ability (WUA)

The equilibrium water uptake ability was determined after immersion of the pre-weighted dry samples in phosphate buffered saline (PBS) 0.1 M pH = 7.4, at 37 °C for 20 s. The weight of the wet samples was measured after PBS excess removal. Then, the WUA was calculated according to the following equation:

(2) $$\mathrm{WUA}\;=\;\frac{Ww-Wd}{Wd}$$

where *Ww* and *Wd* represent the weight of the wet and dry sample, respectively. The process was repeated in triplicate and data were reported as mean and standard deviation. 

### 4.8. Gelatin Release

Gelatin release in NaCl 0.9% solution at 37 °C was determined at increasing time, from 2 to 70 days by colorimetric method using a bicinchoninic acid protein assay (Sigma Chemicals, St. Louis, MO, USA) [56]. 

### 4.9. Strontium Release

For the evaluation of strontium release kinetics, samples G_0.1_HA/G_0.2, G_0.1_HA and S_0.1_HA were weighted, immersed in 5 mL of saline solution (NaCl 0.9% in ultrapure water, added with sodium azide in order to prevent microbial contamination) and stored at 37 °C for different periods of time up to 7 days. At the end of every selected time the medium was completely removed and replaced with 5 mL of saline solution. Three different samples for each time were analyzed. Aliquots of the collected medium were suitably diluted with HNO_3_ ultrapure 0.5 M, containing 10% (*w*/*v*) of LaCl_3_∙H_2_O (99% trace metal basis, Sigma Aldrich). Quantitative determination of strontium content was made by means of atomic absorption spectrophotometer (AAS, Analyst 400, Perkin Elmer Italia, Milano, Italy) equipped with an air-acetylene burner and a strontium lamp working at a wavelength of 460.73 nm. The standard additions method was used.

### 4.10. Statistical Analysis

Statistical evaluation of data was performed using the software package SPSS/PC^+^ Statistics 23.0 (SPSS Inc., Chicago, IL, USA). The results presented are the mean of six independent values. Data are reported as mean ± standard deviations (SD) at a significance level of *p* < 0.05. After having verified normal distribution and homogeneity of variance, a one-way ANOVA was done for comparison between groups. Finally, a post hoc multiple comparison test was performed to detect significant differences among groups. 

## Figures and Tables

**Table 1:** Composition of the obtained (**a**) single-layer and (**b**) double-layer scaffolds.

**Single-Layer Scaffolds**	**% of Genipin (w of Genipin/w of Gelatin)**	
S_0.1_Sr-HA	0.1	
G_0.05	0.05	
G_0.1	0.1	
G_0.15	0.15	
G_0.2	0.2	
G_0.1_Sr-HA	0.1	
(**a**)		
**Double-Layer Scaffolds**	**% of Genipin (w of Genipin/w of Gelatin)**	
	**Inner Layer**	**Outer Layer**
G_0.05/G_0.2	0.05	0.2
G_0.1/G_0.2	0.1	0.2
G_0.15/G_0.2	0.15	0.2
G_0.1_Sr-HA/G_0.2	0.1	0.2
(**b**)		

Samples were labeled according to their genipin content, expressed as ratio (%) between the weight of genipin and gelatin. G refers to rolled scaffolds, while S_0.1_Sr-HA is the single flat layer (non-rolled) scaffold used as reference.

**Table 2:** Extent of crosslinking (%) ^1^.

Sample	(%)
G_0.05	8 ± 1
G_0.1	14 ± 3
G_0.15	26 ± 2
G_0.2	30 ± 1
G_0.1_Sr-HA	15 ± 3

^1^ Each value corresponds to the average of three determinations and it is reported with its own standard deviation.

**Table 3:** Water Uptake Ability ^1^.

Sample	WUA (g PBS/g Gelatin)
S_0.1-Sr-HA	1.7 ± 0.3
G_0.05	3.1 ± 0.9
G_0.1	2.2 ± 0.8
G_0.15	2.2 ± 0.3
G_0.2	2.1 ± 0.4
G_0.1_Sr-HA	1.8 ± 0.3
G_0.1/G_0.2	5.0 ± 0.6
G_0.15/G_0.2	6.9 ± 0.7
G_0.1_Sr-HA/G_0.2	5.0 ± 0.4

^1^ Each value in the table corresponds to the average of three determinations and it is reported with its own standard deviation.

**Table 4:** Gelatin release (w %) ^1^.

Sample	Time (Days)						
	2 Days	7 Days	14 Days	21 Days	28 Days	49 Days	70 Days
S_0.1_Sr-HA	14.8 ± 0.1	22.7 ± 0.1	33.7 ± 2	39.3 ± 1.3	49 ± 2	dissolved	dissolved
G_0.05	26 ± 2	47 ± 2	dissolved	dissolved	dissolved	dissolved	dissolved
G_0.1	16 ± 1	26.9 ± 0.9	36.8 ± 0.4	44.3 ± 0.8	52.1 ± 0.6	dissolved	dissolved
G_0.15	12 ± 1	20.7 ± 1	28.1 ± 0.4	34.6 ± 0.2	40.9 ± 0.2	66 ± 1	dissolved
G_0.2	11 ± 1	17.6±0.8	23.7 ± 0.9	28.7 ± 0.5	32.7 ± 0.6	38.6 ± 0.6	56 ± 2
G_0.1_Sr-HA	16.5 ± 1	27 ± 0.6	36.5 ± 0.8	44 ± 1	52.8 ± 0.5	dissolved	dissolved
G_0.05/G_0.2	22 ± 1	39 ± 1	44 ± 1	50 ± 2	54 ± 2	60 ± 2	dissolved
G_0.1/G_0.2	14.7 ± 0.8	24 ± 1	34 ± 2	42 ± 1	49 ± 1	61 ± 1	dissolved
G_0.15/G_0.2	11 ± 0.6	20 ± 0.8	25 ± 0.8	34 ± 1	42 ± 2	50 ± 2	65 ± 2
G_0.1_Sr-HA/G_0.2	15 ± 1	24.3 ± 0.6	33 ± 1.4	40.8 ± 2	48.3 ± 1	61.3 ± 1	dissolved

^1^ Each value is the average of three determinations and is reported with its own standard deviation.

## References

[1] Petite H., Viateau V., Bensaid W., Meunier A., De Pollak C., Bourguignon M. (2000). Tissue-engineered bone regeneration. Nat. Biotechnol..

[2] Bongio M., van den Beucken J.J., Leeuwenburgh S.C., Jansen J.A. (2010). Development of bone substitute materials: From ‘biocompatible’ to ‘instructive’. J. Mater. Chem..

[3] Langer R., Vacanti J.P. (1993). Tissue engineering. Science.

[4] Wubneh A., Tsekoura E.K., Ayranci C., Uludağ H. (2018). Current state of fabrication technologies and materials for bone tissue engineering. Acta Biomaterialia.

[5] Vallet-Regí M., Colilla M., Gonzalez B. (2011). Medical applications of organic-inorganic hybrid materials within the field of silica-based bioceramics. Chem. Soc. Rev..

[6] Lu L., Zhang X., Wang Y., Ortiz L., Mao X., Jiang Z., Xiao Z., Huang N. (2013). Nanofiber scaffold with gradients in mineral content for spatial control of osteogenesis. ACS Appl. Mater. Interfaces.

[7] Babaie E., Bhaduri S.B. (2018). Fabrication Aspects of Porous Biomaterials in Orthopedic Applications: A Review. ACS Biomater. Sci. Eng..

[8] Darder M., Aranda P., Ferrer M.L., Gutiérrez M.C., Del Monte F., Ruiz-Hitzky E. (2011). Progress in bionanocomposite and bioinspired foams. Adv. Mater..

[9] Soundarya S.P., Haritha Menon A., Viji Chandran S., Selvamurugan N. (2018). Bone tissue engineering: Scaffold preparation using chitosan and other biomaterials with different design and fabrication. Int. J. Biol. Macromol..

[10] Mano J.F., Silva G.A., Azevedo H.B., Malafaya P.B., Sousa R.A., Silva S.S., Boesel L.F., Oliveira J.M., Santos T.C., Marques A.P. (2007). Natural origin biodegradable systems in tissue engineering and regenerative medicine: Present status and some moving trends. J. R. Soc. Interfaces.

[11] Ferreira A.M., Gentile P., Chiono V., Ciardelli G. (2012). Collagen for bone tissue regeneration. Acta Biomaterialia.

[12] Gorgieva S., Kokol V. (2011). Collagen- vs. Gelatine-Based Biomaterials and Their Biocompatibility: Review and Perspectives. Biomater. Appl. Nanomed. IntechOpen.

[13] Veis A. (1964). The Macromolecular Chemistry of Gelatin.

[14] Bigi A., Panzavolta S., Rubini K. (2004). Relationship between triple helix content and mechanical properties of gelatin films. Biomaterials.

[15] Gòmez-Guillén M.C., Giménez B., Lòpez-Caballero M.E., Montero M.P. (2011). Functional and bioactive properties of collagen and gelatin from alternative sources: A review. Food Hydrocoll..

[16] Lien S.M., Ko L.Y., Huang T.J. (2009). Effect of pore size on ECM secretion and cell growth in gelatin scaffold for articular cartilage tissue engineering. Acta Biomater..

[17] Rault J., Herbage F.D., Abdul-Malak N., Huc A. (1996). Evaluation of different chemical methods for crosslinking collagen gels, films and sponges. J. Mater. Sci. Mater. Med..

[18] Sung H.W., Liang I.L., Chen C.N., Huang R.N., Liang H.F. (2011). Stability of a biological tissue fixed with a naturally occurring cross-linking agent (genipin). J. Biomed. Mater. Res..

[19] Bigi A., Cojazzi G., Panzavolta S., Roveri N., Rubini K. (2002). Stabilization of gelatin films by crosslinking with genipin. Biomaterials.

[20] Yao C.H., Liu B.S., Chang C.J., Hsu S.H., Chen Y.S. (2004). Preparation of networks of gelatin and genipin as degradable biomaterials. Mater. Chem. Phys..

[21] Huang K.S., Lu K., Yeh C.S., Chung S.R., Lin C.H., Yang C.H., Dong Y.S. (2009). Microfluidic controlling monodisperse microdroplet for 5-fluorouracil loaded genipin-gelatin microcapsules. J. Control. Release.

[22] Deeks E.D., Dhillon S. (2010). Strontium ranelate: A review on its use in the treatment of postmenopausal osteoporosis. Drugs.

[23] Gallacher S.J., Dixon T. (2010). Impact of treatments for postmenopausal osteoporosis (bisphosphonates, parathyroid hormone, strontium ranelate and denosumab) on bone quality: A systematic review. Calcif. Tissue Int..

[24] Marie P.J. (2005). Strontium ranelate: A novel mode of action optimizing bone formation and resorption. Osteoporosis Int..

[25] Ammann P. (2005). Strontium ranelate: A novel mode of action leading to renewed bone quality. Osteoporosis Int..

[26] Reginster J.Y., Bruyère O., Sawicki A., Roces-Varela A., Fardellone P., Roberts A., Devogelaer J.P. (2009). Long-term treatment of postmenopausal osteoporosis with strontium ranelate: Results at 8 years. Bone.

[27] Saidak Z., Marie P.J. (2012). Strontium signaling: Molecular mechanisms and therapeutic implications in osteoporosis. Pharmacol. Ther..

[28] Panzavolta S., Torricelli P., Casolari S., Parrilli A., Fini M., Bigi A. (2018). Strontium- substituted hydroxyapatite- gelatin biomimetic scaffolds modulate bone cell response. Macromol. Biosci..

[29] Panzavolta S., Torricelli P., Sturba L., Bracci B., Giardino R., Bigi A. (2007). Setting properties and in vitro bioactivity of strontium-enriched gelatin–calcium phosphate bone cements. J. Biomed. Mater. Res. A.

[30] Bracci B., Torricelli P., Panzavolta S., Boanini E., Giardino R., Bigi A. (2009). Effect of Mg^2+^, Sr^2+^ and Mn^2+^ on the chemico-physical and in vitro biological properties of calcium phosphate biomimetic coatings. J. Inorg. Biochem..

[31] Surmenev R.A., Surmeneva M.A., Ivanova A.A. (2014). Significance of calcium phosphate coatings for the enhancement of new bone osteogenesis: A review. Acta Biomater..

[32] Graziani G., Bianchi M., Sassoni E., Russo A., Marcacci M. (2017). Ion-substituted calcium phosphate coatings deposited by plasma-assisted techniques: A review. Mater. Sci. Eng. C.

[33] Tadier S., Bareille R., Siadous R., Marsan O., Charvillat C., Cazalbou S., Amédé J., Rey C., Combes C. (2012). Strontium-loaded mineral bone cements as sustained release systems: Compositions, release properties, and effects on human osteoprogenitor cells. J. Biomed. Mater. Res. B.

[34] Ginebra M.P., Canal C., Espanol M., Pastorino D., Montufar E.B. (2012). Calcium phosphate cements as drug delivery materials. Adv. Drug Deliv. Rev..

[35] Weiner S., Wagner H.D. (1998). The material bone: Structure-mechanical function relations. Annu. Rev. Mater. Sci..

[36] Jiang H., Zou Y., Wang H., Du J., Li Y., Yang X. (2013). Biomimetic spiral- cylindrical scaffold based on hybrid chitosan/cellulose/nano-hydroxyapatite membrane for bone regeneration. ACS App. Mater. Interfaces.

[37] Wan Y., Feng G., Shen F.H., Laurencin C.T., Li X. (2008). Biphasic scaffold for annulus fibrosus tissue regeneration. Biomaterials.

[38] Light N.D., Macgregor J., Harvey W.W., Paul W. (1996). Absorbable Structures for Ligament and Tendon Repair. U.S. Patent.

[39] Berman A.B. (2000). Resorbable Interposition Arthroplasty Implant. U.S. Patent.

[40] Sussman M.B., Zvi Kadouri A. (1993). Fibrous Matrix for in vitro Cell Cultivation. U.S. Patent.

[41] Wang J., Shah A., Yu X. (2011). The influence of fiber thickness, wall thickness and gap distance on the spiral nanofibrous scaffolds for bone tissue engineering. Mater. Sci. Eng. C.

[42] Dahl S.G., Allain P., Marie P.J., Mauras Y., Boivin G., Ammann P., Tsouderos Y., Delmas P.D., Christiansen C. (2001). Incorporation and distribution of strontium in bone. Bone.

[43] Gioffrè M., Torricelli P., Panzavolta S., Rubini K., Bigi A. (2012). Role of pH on stability and mechanical properties of gelatin films. J. Bioact. Compat. Pol..

[44] Okuyama K. (2008). Revisiting the molecular structure of collagen connective tissue. Tissue Res..

[45] Amadori S., Torricelli P., Panzavolta S., Parrilli A., Fini M., Bigi A. (2015). Highly porous gelatin reinforced 3D scaffolds for articular cartilage regeneration. Macromol. Biosci..

[46] Davidenko N., Gibb T., Schuster C., Best S.M., Campbell J.J., Watson C.J., Cameron R.E. (2012). Biomimetic collagen scaffolds with anisotropic pore architecture. Acta Biomater..

[47] Panzavolta S., Torricelli P., Amadori S., Parrilli A., Rubini K., della Bella E., Fini M., Bigi A. (2013). 3D interconnected porous biomimetic scaffolds: In vitro cell response. J. Biomed. Mater. Res. A.

[48] Panzavolta S., Fini M., Nicoletti A., Bracci B., Rubini K., Giardino R., Bigi A. (2009). Porous composite scaffolds based on gelatin and partially hydrolyzed α-tricalcium phosphate. Acta Biomater..

[49] Walther A., Bjurhager I., Malho J.M., Pere J., Ruokolainen J., Berglund L.A., Ikkala O. (2010). Large-area, lightweight and thick biomimetic composites with superior material properties via fast, economic, and green pathways. Nano Lett..

[50] Miles C.A., Ghelashvili M. (1999). Polymer-in-a-box mechanism for the thermal stabilization of collagen molecules in fibers. Biophys. J..

[51] Goldstein S.A. (1987). The mechanical properties of trabecular bone: Dependence on anatomic location and function. J. Biomech..

[52] Homminga J., McCreadie B.R., Ciarelli T.E., Weinans H., Goldstein S.A., Huiskes R. (1987). Cancellous bone mechanical properties from normals and patients with hip fractures differ on the structure level, not on the bone hard tissue level. Bone.

[53] Giesena E.B.W., Dingb M., Dalstrab M., van Eijdena T.M.G.J. (2001). Mechanical properties of cancellous bone in the human mandibular condyle are anisotropic. J. Biomech..

[54] Amadori S., Torricelli P., Panzavolta S., Parrilli A., Fini M., Bigi A. (2015). Multi-layered scaffolds for osteochondral tissue engineering: In vitro response of co-cultured human mesenchymal stem cells. Macromol. Biosci..

[55] Tanimoto Y., Hayakawa T., Sakae T., Nemoto K. (2005). Characterization and bioactivity of tap-cast and sintered TCP Sheets. J. Biomed. Mater. Res. Part A.

[56] Hataka T., Sato H., Watanabe Y., Matsumoto M. (1994). Effect of formaldehyde on the physiochemical properties of soft gelatin capsule shells. Chem. Pharm. Bull..

